# High resolution structural and functional analysis of a hemopexin motif protein from *Dolichos*

**DOI:** 10.1038/s41598-019-56257-6

**Published:** 2019-12-27

**Authors:** Sarita Chandan Sharma, Ashish Kumar, Sharad Vashisht, Dinakar M. Salunke

**Affiliations:** 1 Regional Centre for Biotechnology, NCR Biotech Science Cluster, Faridabad, 121001 India; 2 Manipal Academy of Higher Education, Madhav Nagar, Manipal, Karnataka 576104 India; 3 International Centre for Genetic Engineering and Biotechnology, New Delhi, 110067 India

**Keywords:** Biophysics, X-ray crystallography

## Abstract

It is increasingly evident that seed proteins exhibit specific functions in plant physiology. However, many proteins remain yet to be functionally characterized. We have screened the seed proteome of *Dolichos* which lead to identification and purification of a protein, DC25. The protein was monomeric and highly thermostable in extreme conditions of pH and salt. It was crystallized and structure determined at 1.28 Å resolution using x-ray crystallography. The high-resolution structure of the protein revealed a four-bladed β-propeller hemopexin-type fold containing pseudo four-fold molecular symmetry at the central channel. While the structure exhibited homology with 2S albumins, variations in the loops connecting the outermost strands and the differences in surface-charge distribution may be relevant for distinct functions. Comparative study of the protein with other seed hemopexins revealed the presence of four conserved water molecules in between the blades which cross-link them and maintain the tertiary structure. The protein exhibited intrinsic peroxidase activity, which could be inhibited by binding of a heme analog. The identification of redox-sensitive cysteine and inhibition of peroxidase activity by iodoacetamide facilitated characterization of the possible active site. The determined peroxidase activity of DC25 may be responsible for rescuing germinating seeds from oxidative stress.

## Introduction

Seeds have a good storage of carbohydrates, fats, and proteins. Proteins constitute approximately 10 to 40% of seed dry weight in cereals, legumes and oilseeds. The seed proteins have been segregated on the basis of solubility into albumins, globulins, prolamines, and glutelins^1^. Earlier, it was believed that seed proteins, which are mainly composed of nitrogen, degrade to supply nutrition to developing embryo during germination. However, it was later found that mostly vicilins and legumins degrade since they are localized in membrane-bound vesicles^2,3^ whereas albumins and lectins remain longer^4^ since they are present in the cytoplasm^5^. Some seed proteins are known as biologically active viz., lectins, enzymes and enzyme inhibitors, and play their functional roles in different pathways of plant physiology^6^.

Seed is a complex plant organ, which by itself is an embryonic plant in the inactive state. The organ has low concentration of oxygen, i.e., it is in hypoxic condition. As the seed starts to absorb water, the germination process begins which relieves the seed from desiccation and hypoxia but it is accompanied with the release of reactive oxygen species (ROS) among other by-products^7^. The superoxide anion is converted to H_2_O_2_ by superoxide dismutase and later, detoxified to water by peroxidases. At moderate concentrations, ROS have a physiological role in a number of plant signaling processes, such as photosynthesis and respiration regulation, cellular metabolic fluxes and plant organogenesis^8,9^. However, at a higher level, ROS leads to molecular oxidation causing malfunction at the cellular level and eventual cell death. In this situation, antioxidant compounds and enzymes like thioredoxin peroxidase and catalase rescue the cells from damage by neutralizing and detoxifying hydrogen peroxide or organic hydroperoxides^10^.

The germination rate has an interesting relationship with the production of antioxidant compounds and an increase in enzyme activities. Increase of ascorbate and reduced-glutathione was reported during seed imbibitions^11,12^. On the other hand, α-tocopherol^13^, flavonoids and phenolics^14,15^ increase during germination. In a similar fashion, the enzymatic activity of glutathione reductase, catalase^16^ and superoxidase dismutase^17^ increase during seed imbibitions and germination. These changes provide evidence supporting ROS scavenging for successful seed germination.

Legume plants are being cultivated for many years for food and animal feed. Their seeds contain about 20,000 different proteins^18^ which play structural and metabolic roles including a few storage proteins which are destined to provide amino acids and nitrogen during seed germination. *Dolichos* cultivated worldwide for human consumption is one among them. The protein content of *Dolichos* seeds varies from 20–28% and a few of these proteins, e.g. protease inhibitor^19^, mannose/glucose-binding lectin^20^ and α-mannosidase^21^, have been greatly studied. However, many proteins remain yet to be fully characterized.

Here we report the structure-function characterization of DC25, a protein from albumin fraction of *Dolichos*. The significant differences could be observed with respect to other members of 2S albumin in the conformation of loops connecting the outermost strands of the blades and in the surface-charge distribution, while the core structure is highly conserved with respect to the previously reported hemopexins. We have, for the first time, established inherent peroxidase activity of DC25. A heme analog molecule was shown to bind to DC25 effectively reducing its peroxidase activity. Our study shed light on the physiological role of DC25, acting as a detoxifying agent to relieve the germinating seed from oxidative stress.

## Results

### Fractionation and characterization of proteins from *Dolichos* seeds

**Figure 1:**
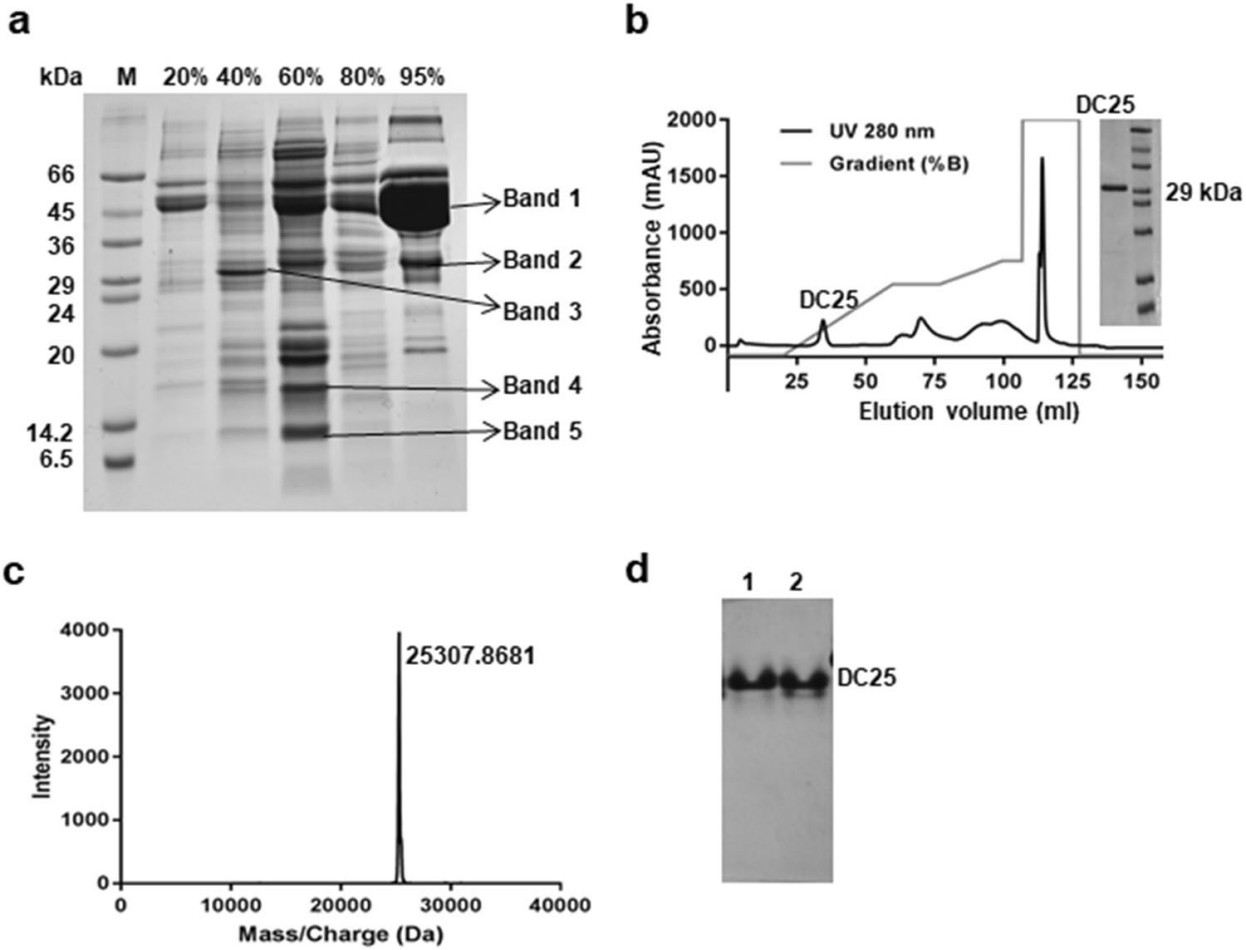
Purification and characterization of DC25. (**a**) Lane M corresponds to marker proteins and subsequent lanes for salt fractions from 20% to 95%. (**b**) Weak anion exchange chromatogram and 15% SDS-PAGE showing presence of pure DC25. (**c**) Intact mass analysis of purified DC25. (**d**) The 10% native gel profile of native (lane 1) and heat treated (lane 2) of DC25.

The different fractions obtained after ammonium sulfate precipitation were characterized by 15% SDS-PAGE (Fig. 1a). The N-terminal sequences of the bands 1, 4, and 5 showed homology to different seed proteins (Supplementary Table S1). Band 1 showed homology to vicilin from *Vigna unguiculata*, band 4 showed homology to mannose lectin from *Lablab purpureus* and band 5 showed homology to a legume lectin.

The N-terminal sequencing of band 2 and band 3 was not successful. However, the internal peptide fragments of bands 2 and 3 showed homology with the known proteins (Supplementary Table S1). In the case of band 2, the generated peptides showed homology with mung bean seed albumin. It has been named as DC25 as it has been purified from *Dolicos* and has 25 kDa molecular weight. The peptides from band 3 were homologous with dehydrin from *Vigna unguiculata*.

The weak anion exchange chromatography of 95% ammonium sulfate fraction gave a peak at 60 mM NaCl in the elution buffer. The SDS-PAGE analysis indicated the purity of the protein (Fig. 1b). Matrix-assisted laser desorption ionization (MALDI) analysis of DC25 showed its molecular weight as 25,307.89 Da (Fig. 1c). 10% native gel profile of DC25 without heating and after mild heating showed a single band at the same position, which indicated the presence of a homogenous monomeric protein (Fig. 1d).

Measurement of melting temperature indicates the inherent thermal stability of the protein. The thermal shift assay (TSA) analysis of DC25 was done using different concentrations of sodium chloride at a pH range of 2.2 to 11. This analysis suggested that DC25 is a highly stable protein even at low pH of 2.2 and a high pH of 11 in the presence of 60 mM, 500 mM, and 1 M NaCl (Supplementary Fig. S1).

### Crystallization and structure determination

The purified DC25 was crystallized as rod-shaped crystals through hanging drop vapor diffusion method at room temperature using protein concentration of 11 mg/ml at pH 4.6 in 0.1 M sodium acetate buffer and 2 M ammonium sulfate as precipitant. The diffraction data were recorded at 1.28 Å resolution on synchrotron radiation source (BM14, ESRF) and processed using HKL2000^22^ which revealed that the crystals belonged to the space group P3_1_. The data statistics are given in Table 1. The Matthews coefficient was 1.93 Å^3^Da^−1^ that indicated the presence of three monomers of DC25 in one asymmetric unit.

**Table 1:** Data collection statistics: Values in parentheses are for the highest resolution shell. R_merge_ = Σ|I − < I > |/ΣI, where I is the integrated intensity of a given resolution shell.

Data collection	
Diffraction source	BM14, ESRF
Wavelength (Å)	0.88560
Space group	P3_1_
a, b, c (Å)	65.2, 65.2, 118.2
α, β, γ (°)	90.0, 90.0, 120.0
Resolution range (Å)	31.41-1.28 (1.29-1.28)
Total No. of reflections	389773 (27519)
No. of unique reflections	140457 (13099)
Completeness (%)	97 (90.1)
Multiplicity	2.8 (2.1)
Mean I/σ(I)	15.6 (2.6)
R _merge_	0.054 (0.432)
**Refinement**	
R factor	0.13
R _free_	0.17
Average B-factor (Å^2^) for Protein	10.9
Average B-factor (Å^2^) for Solvent	23.7
Average B-factor (Å^2^) for Ligand	14.6
RMSD (Bond length) (Å)	0.020
RMSD (Bond angle) (°)	2.169
Ramachandran plot favored (%)	99.5
Ramachandran plot disallowed (%)	0.5

Refinement statistics: R_work_ = Σ||F_obs_| − |F_cal_||/Σ|F_obs_|.

The identified peptides showed homology with CP4 protein after BLAST, hence CP4 was used as a model for molecular replacement structure determination which produced initial phasing statistics with log-likelihood gain (LLG), rotation factor Z score (RFZ) and translation factor Z score (TFZ) were 24884, 11.7 and 83.6 respectively. The initial R_factor_ was 29.3% and R_free_ was 31.76%, which were refined to 12.84% and 16.98% respectively. The 99.53% residues were in the allowed region of the Ramachandran plot after successive iterative refinement cycles. The refinement statistics are given in Table 1.

### Overall structure of DC25

The final structure of DC25 is now deposited in the protein data bank (PDB ID: 6IX1). The structure shows three monomers per asymmetric unit. Each monomer has a β-propeller domain, typical of the hemopexin superfamily proteins. It looks like a disc-shaped structure, with an average diameter of 41 Å and a thickness of 26 Å. It has a pseudo-4-fold axis, which passes through the molecular center, defined by a channel. The protein has solvent accessible surface area of 17624.22 Å^2^.

**Figure 2:**
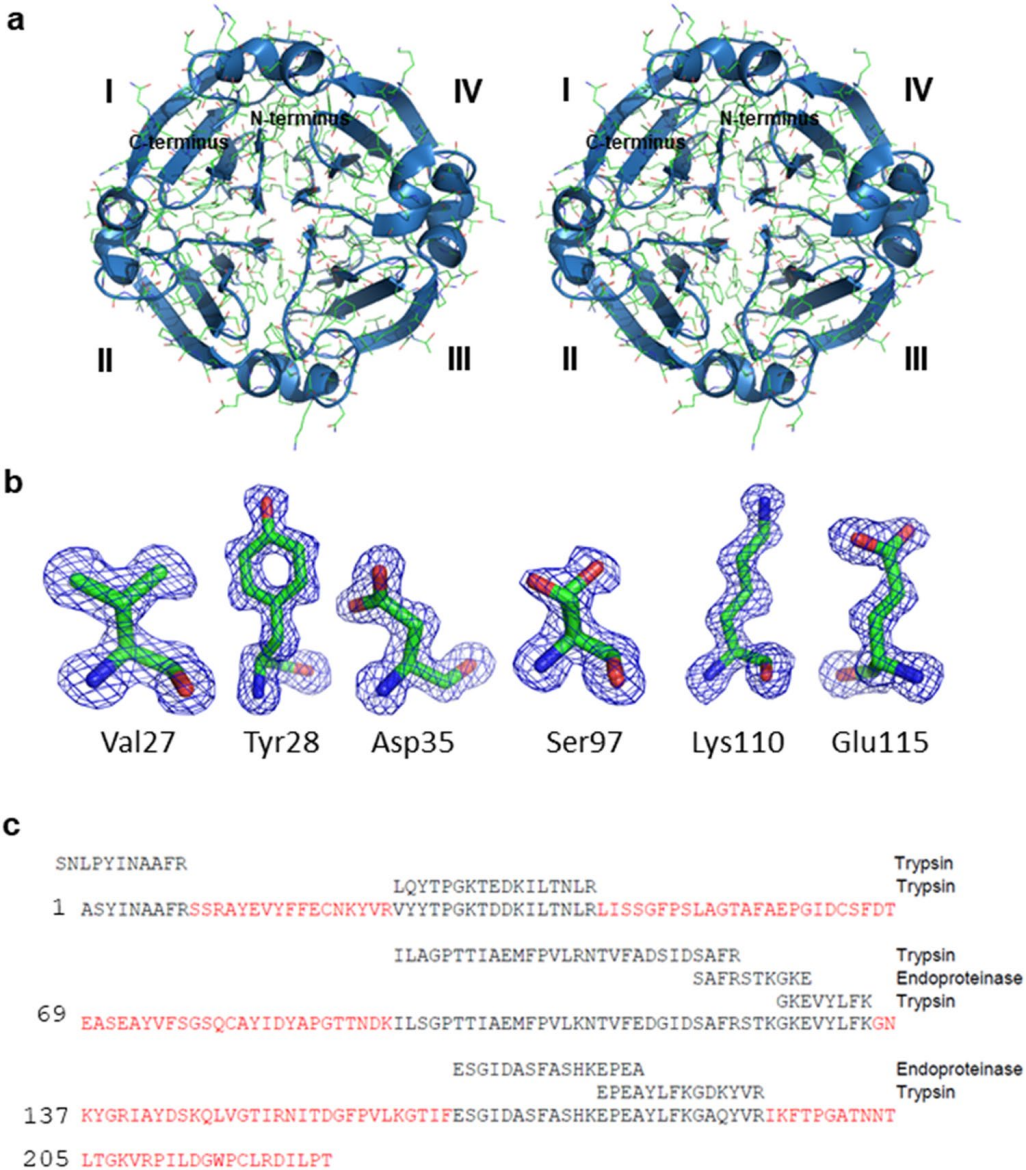
Overall structure and complete sequence of DC25. (**a**) Stereo view of ribbon diagram showing β-propeller fold of DC25. (**b**) The polder omit map (blue mesh) of Val27, Tyr28, Asp35, Ser97, Lys110 and Glu115 of chain A contoured at 3σ have been calculated using Polder Maps in PHENIX^45^. The residues are shown as stick in elemental colors and the figure was rendered using PyMOL^26^ (The PyMOL Molecular Graphics System; http://www.pymol.Org). Ser97 is present in dual conformation with half occupancy in chain A. (**c**) DC25 sequence derived on the basis of internal sequencing and the interpretation of electron density map calculated using phases at 1.28 Å resolution. The internal sequences homologous to mung bean seed albumin obtained by proteolysis using trypsin and endoproteinase Glu-C have been aligned. The portions of the sequence interpreted from electron density map are highlighted in dark red.

The β-propeller domain is formed by circularly arranged four blades around the channel perpendicular to the plane of the blades. Each blade is composed of a β-sheet consisting of four antiparallel β-strands. In many cases, the four-bladed propeller structure helps in substrate binding. Blade I of the β-propeller domain, which is towards the N-terminus is in close proximity to the blade IV, which is towards the C-terminus end. The strands near the central channel are better aligned compared to the outer ones. The adjacent blades are linked by two short α-helices (Fig. 2a).

The central channel consisted of four innermost β-strands of the blades, which are arranged in a parallel manner. It looks like an inverted funnel having a narrow diameter of 6.7 Å at the N-terminal end and the wider end diameter is 8.3 Å. The narrow end of the channel is lined with acidic amino acids, while the wider end is lined with basic amino acids. In the case of Blade I, asparagine is the first internal residue of innermost strand forming the channel. On the other hand, aspartic acid is the first residue in the case of remaining three blades. The four blades of DC25 align very nicely between themselves with root mean square deviations (RMSD) ranging from 0.4 Å to 0.8 Å. In the case of mammalian origin hemopexins the variations among different blades are much higher. There is a presence of bulge in the case of blade III and IV due to disrupted conformation of the β-strand.

Apart from obtaining DC25 sequences by protein sequencing method, remaining protein sequence and the ambiguities in the identified sequence, were resolved by interpretation of the electron density map at 1.28 Å resolution during iterative refinement. The polder map of six representative residues, namely, Val27, Tyr28, Asp35, Ser97, Lys110, and Glu115 are shown in Fig. 2b where Ser97 is present in dual conformation with half occupancy in chain A. The CC(1,3) values for these residues are higher than those of CC(1,2) and CC(2,3) which indicate the presence of dominant electron density for the concerned residues.The packing of three molecules of DC25 has been shown in two unit cells (Supplementary Fig. S2). Combined data consisting of mass spectrometry and high resolution electron density map enabled resolving the complete sequence of DC25 (Fig. 2c).

### Structural comparison with other seed hemopexins

**Figure 3:**
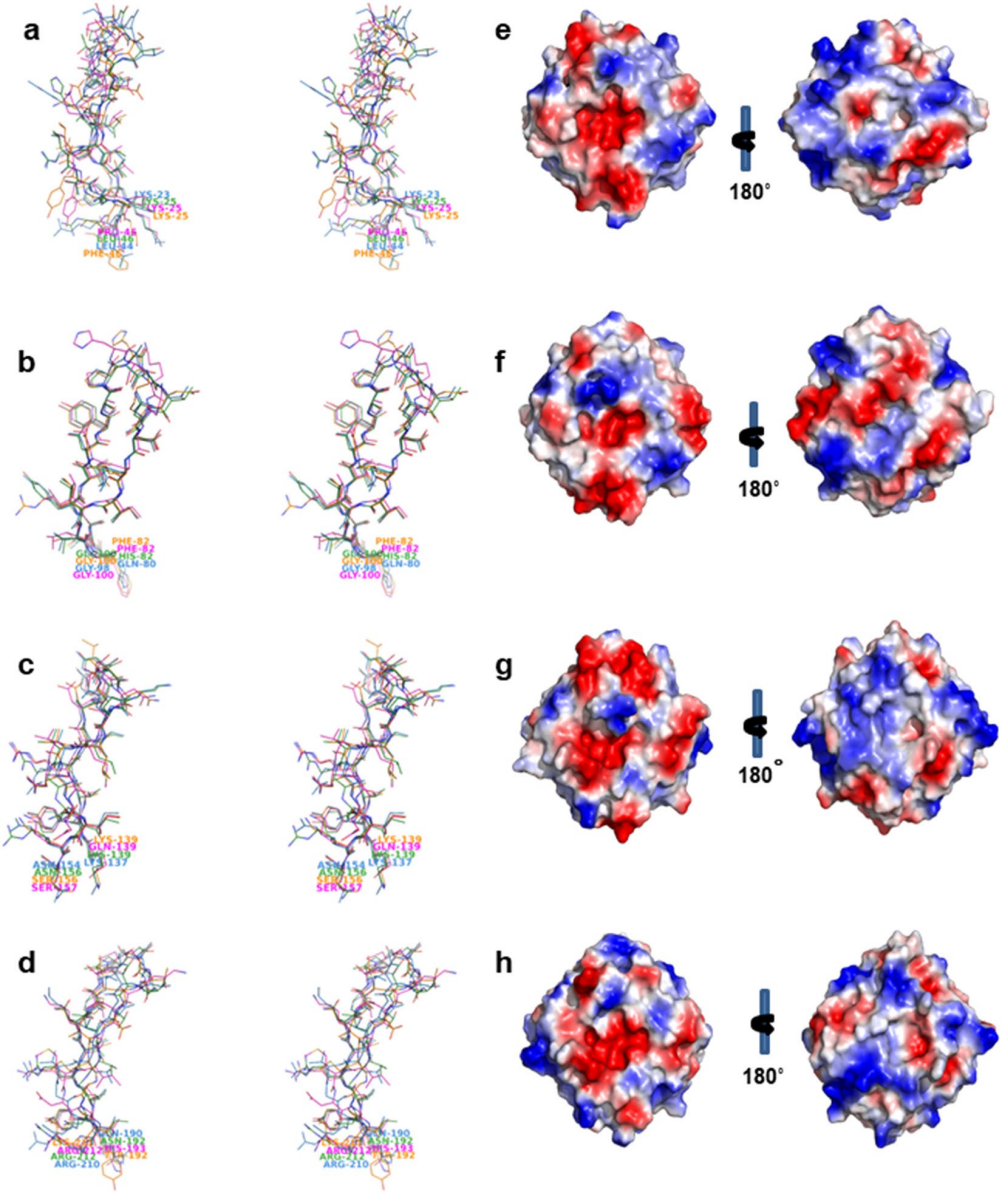
Stereo view of the conformational differences in the loops and surface charge distribution of hemopexin proteins. Panels a to d correspond to blade I to IV of DC25 (skyblue), CP4 (green), LS24 (magenta) and CAL (orange). Panels e to h correspond to surface charge distribution of DC25 (e), CP4 (f), LS24 (g) and CAL (h). The figure was prepared using PyMOL^26^ (The PyMOL Molecular Graphics System; http://www.pymol.Org).

While DC25 shows substantial differences with respect to the mammalian hemopexins, it has significant conformational similarity with the hemopexin fold proteins from plant sources. Crystal structures of only three other proteins from plants exhibiting hemopexin fold belonging to 2S albumin family have been determined. The structures of CP4^23^ (PDB ID: 3OYO), LS24^24^ (PDB ID: 3LP9) and CAL^25^ (PDB ID: 3V6N) have been determined at the resolution of 2.1 Å, 2.2 Å and 2.2 Å, respectively. The structure of DC25 determined is at the highest resolution of 1.28 Å and provided better structural insights. It provided avenues for comparative analyses among the four different 2S albumins. When the monomers of these proteins were superimposed, it was found that the core β-propeller structure was highly superimposable between DC25 and other three hemopexins from plant seeds with C^α^ RMSD values in the range of 0.32 Å to 0.54 Å. Effectively, differences in the sequences between the four proteins did not have significant consequences for their conformations. However, the exposed regions of the proteins show differences with RMSD values up to about 1.87 Å (Fig. 3a,b,c,d). The positively charged and negatively charged amino acids of the protein are not uniformly distributed along the surface, but they are present in positively and negatively charged patches. Mainly the negatively charged residues accumulate around the narrower end and positively charged residues around the wider end of the channel (Fig. 3e,f,g,h).

There are three molecules of DC25 in the asymmetric unit. There is no pseudo three-fold symmetry between the three molecules in the asymmetric unit. The coordinates data was subjected to rotation and translation analysis using PyMOL^26^. It was found that molecule A is rotated and translated along crystallographic a-axis transforming into molecule C with an angle of rotation 178° and the length of the translation vector along the rotation axis is 31 Å. While molecule B showed 13° angle of rotation and 19 Å length of the translation vector along the rotation axis towards a-axis. The way the three molecules are juxtaposed with each other also indicates that symmetric arrangement between them is unlikely. The crystallographic packing diagram is provided as Supplementary Fig. S2.

We have identified 628 water molecules in the asymmetric unit during refinement of DC25 structure, which was possible due to the high-resolution diffraction data. The organization of the water structure with respect to the three molecules of DC25 in the asymmetric unit has been shown in the Supplementary Fig. S3a. The hydration structure of each monomer was similar. However, the non-symmetric arrangement of the monomers in the asymmetric unit of DC25 crystals is such that the monomer B of DC25 is far away from monomer A than monomer C. The presence of five water molecules effectively held these two monomers (A and C) together. Out of these, three water molecules (Wat421, 432, and 508) were associated with chain A and interacted with a stretch of residues (Ser97, Thr125, Pro198, and Asn202). The remaining two water molecules (Wat414 and 424) were associated with chain C and interacted with residues Thr100, Glu104 and Thr125.

The three monomers of DC25, when superimposed, revealed presence of ten conserved water molecules (Supplementary Fig. S3b). When the refined DC25 structure was superimposed on CP4, LS24 and CAL, seven water molecules were found conserved (Supplementary Fig. S3c) among the four hemopexins. In order to refer to the residues and water molecules, the crystal structure of DC25 has been taken as a reference. Out of the 7 conserved water molecules, three water molecules (Wat428, 455, and 520) were present in the cavity whereas remaining four water molecules (Wat429, 437, 487 and 519) were buried and exist between the blade interfaces probably designed to stabilize the ß-propeller architecture.

**Figure 4:**
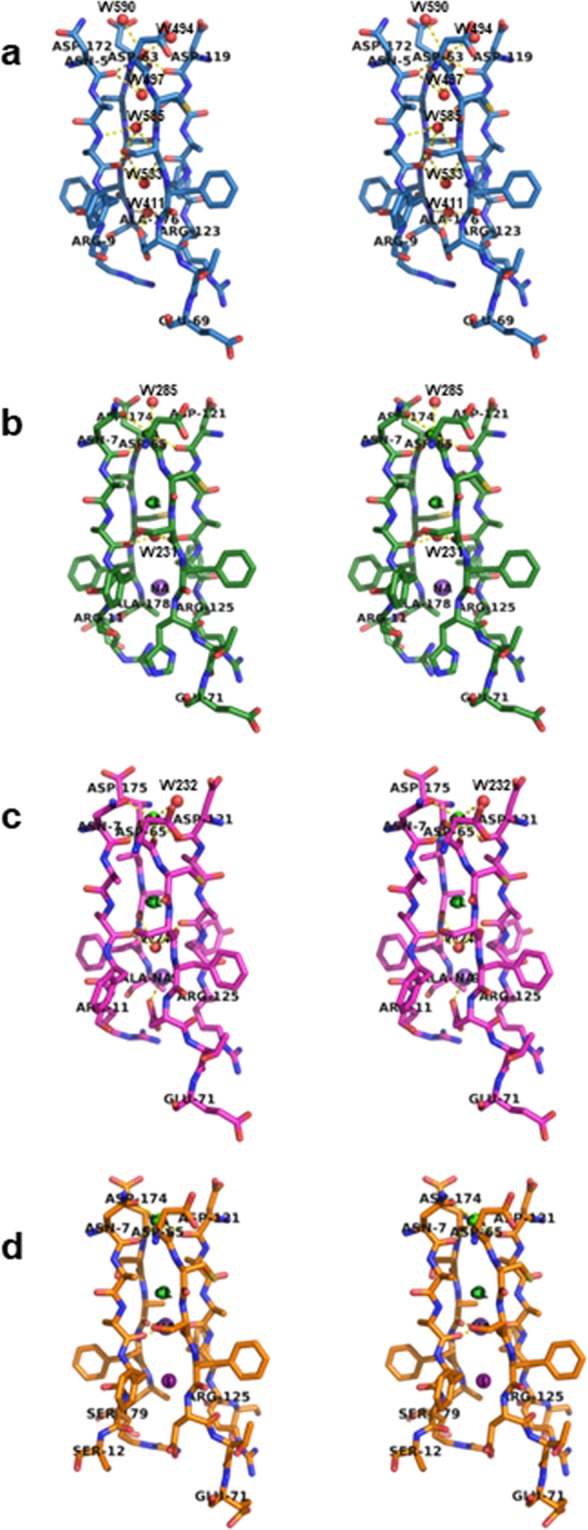
Stereo view of the channels belong to hemopexins in stick. The channel of DC25 was shown in sky blue (**a**), the channel of CP4 was shown in green (**b**), the channel of LS-24 was shown in magenta (**c**) and the channel of CAL was shown in orange (**d**). The figure was prepared using PyMOL^26^ (The PyMOL Molecular Graphics System; http://www.pymol.Org).

The central channel in the case of DC25 appears to be distinct compared with the channels in the other three plant hemopexins. Five water molecules (Wat403, 411, 497, 533 and 585) were present inside the channel of DC25 (Fig. 4a), while only one water molecule and three ions like calcium, chloride, and sodium were present in the case of CP4 and LS24 (Fig. 4b,c). On the other hand, iodine, calcium, chloride and sodium are present in case of CAL without any water molecule (Fig. 4d).

### DC25-Porphyrin binding

**Figure 5:**
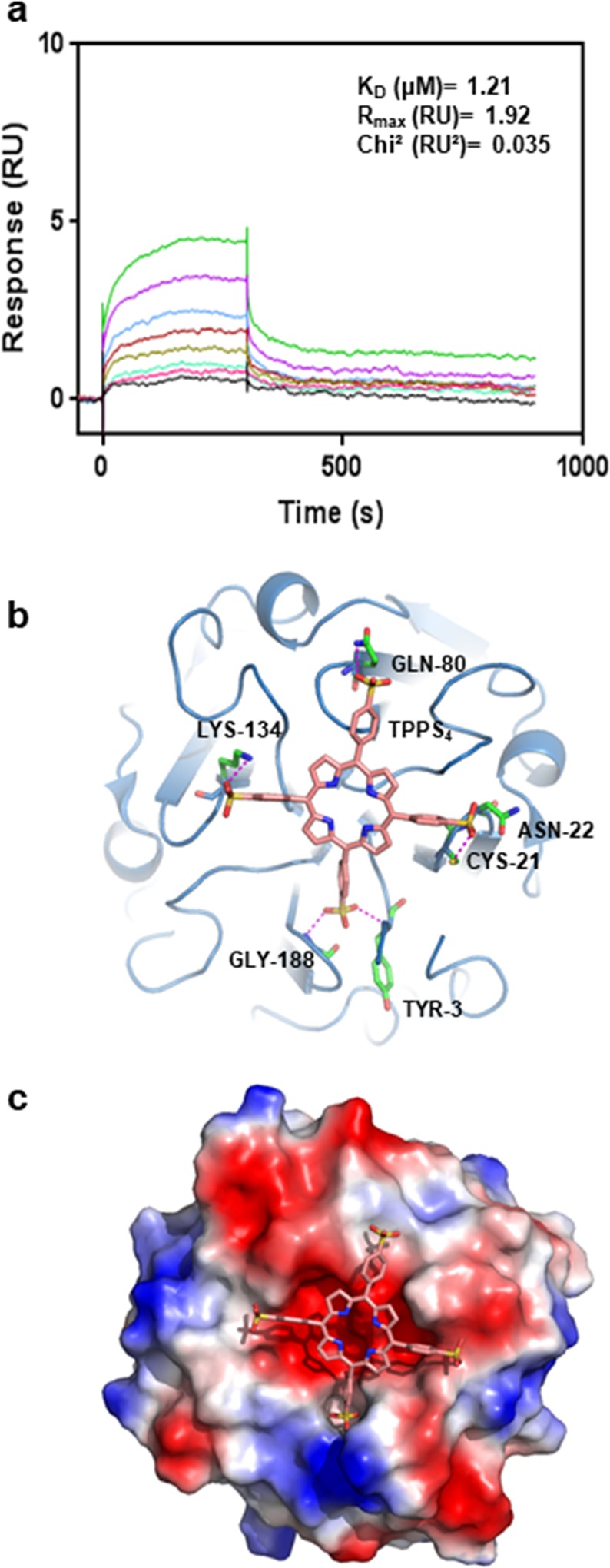
Binding affinity of DC25 with TPPS_4_, and induced-fit docking. (**a**) Analysis of DC25 (ligand) binding affinity with different concentrations of TPPS_4_ (analyte) in range of 0.1 to 12.5 µM by SPR. RU, response units. (**b**) DC25 has been shown in sky blue ribbon whereas docked TPPS_4_ has been shown in a light pink stick. The H-bond interactions (magenta dash lines) between amino acid residues (green) and -SO_4_2^−^ (yellow) of TPPS_4_. (**c**) Electrostatic potential surface view of the docked TPPS_4_ on DC25. The figure was prepared using PyMOL^26^ (The PyMOL Molecular Graphics System; http://www.pymol.Org).

It is well documented that the hemopexins are heme binding proteins. Heme was not soluble in buffer as required for experimental conditions, however heme analog tetrasodium meso-tetra(sulfonatophenyl)porphine dodecahydrate (TPPS_4_) was soluble. Therefore, DC25 was subjected to binding analysis with TPPS_4_ using surface plasmon resonance (SPR). The dose-dependent kinetic analysis of TPPS_4_ binding to DC25 was carried out. Binding was monitored as response units (RU) versus time which was represented as sensorgram. The obtained sensorgrams were fitted using the 1:1 Langmuir binding model, which revealed equilibrium dissociation constant (K_D_) of 1.21 µM (Fig. 5a).

There is no structure of any heme bound protein homologous to DC25 is available, so we tried to co-crystallize DC25 with heme and heme analogs but we could not crystallize the complex. Hence, induced fit docking was performed to understand the potential mode of DC25/TPPS_4_ binding generating 69 poses. Out of these, one pose had highest binding free energy (−6.28 kcal/mol) (Fig. 5b), which supports the observed physiological binding affinity through SPR. The analysis of binding model indicated that ligand binding pocket of DC25 was formed with mainly negatively charged residues (Fig. 5c). The examination of Fig. 5b indicates reasonable complementarity between the ligand and the protein. However, the interacting residues of the protein do not exhibit any four-fold symmetry. The TPPS_4_ with sulphate polar head forms six hydrogen bonds with Tyr3, Cys21, Asn22, Gln80, Lys134 and Gly188 besides hydrophobic interactions with DC25.

### Functional characterization of DC25

**Figure 6:**
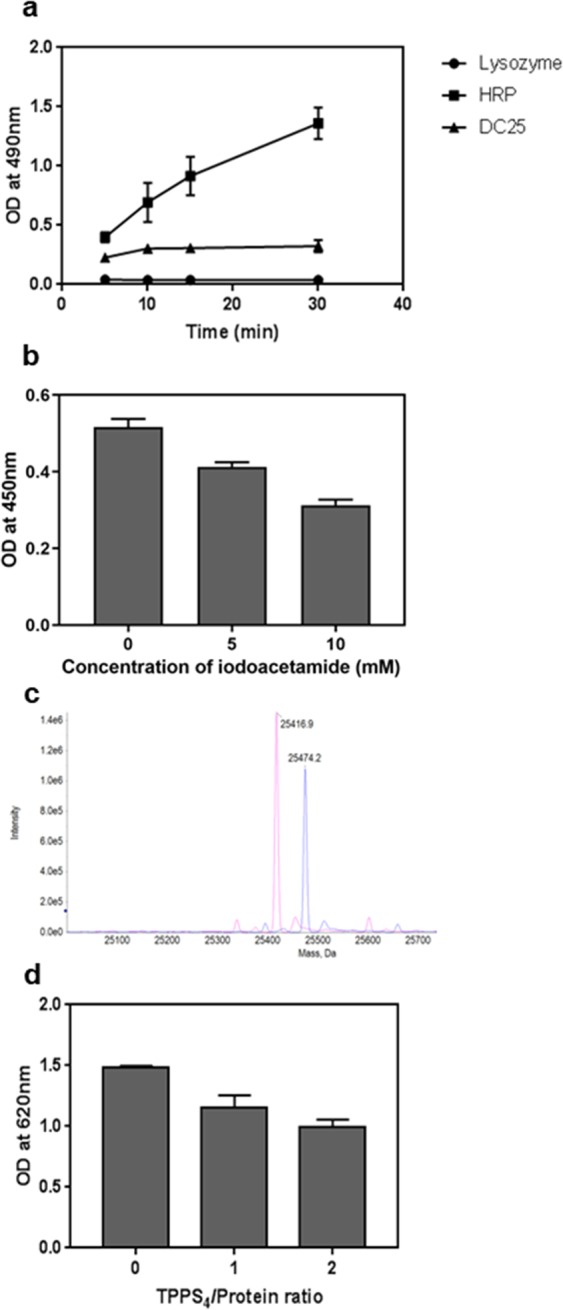
Functional characterization of DC25. (**a**) Time dependent assay to estimate the peroxidase activity of DC25 (**b**) The inhibition assay of peroxidase activity of DC25 by iodoactamide. (**c**) MS spectra after oxidation of the cysteine residue by iodoacetamide showing pink colored peak corresponds to without iodoacetamide treated DC25 whereas blue colored peak corresponds to iodoacetamide treated DC25. (**d**) The inhibition assay of peroxidase activity of DC25 by TPPS_4_.

It is clear that DC25 is homologous to the albumin 2 family proteins. It was known that albumins remain longer during the seed germination process, which provides an indication of the possible physiological role. It was suggested in earlier reports that seven and eight-bladed propeller proteins function as oxidoreductases such as methanol dehydrogenase, obtained from methylotrophic bacteria and catalyze the oxidation of methanol to formaldehyde^27^. Therefore, DC25 was tested for the similar oxidoreductase activity. It showed intrinsic peroxidase activity that is increasing with time (Fig. 6a) and was lower than that of horseradish peroxidase, but still physiologically relevant.

The DC25-docked with TPPS_4_ model showed that Cys21 was interacting with SO_4_^2−^ of TPPS_4_. This residue was also predicted to have highest redox sensitivity potential (Table 2). Therefore, the DC25 was alkylated with different concentrations of iodoacetamide and the peroxidase activity assay was performed. The reduction in peroxidase activity was evident (Fig. 6b). The intact mass of the alkylated protein was determined which showed an increase in mass of 57 Da in comparison to the non-alkylated native protein (Fig. 6c). This clearly suggested alkylation of a single cysteine which is most probably Cys21 that is present on the protein surface since Cys64 having decision value close to Cys21 is buried. In order to explore if any correlation exists between TPPS_4_ binding site with the active site, the peroxidase activity assay was performed in the presence of TPPS_4_ at different ratios of TPPS_4_ to DC25. In other words, peroxidase activity was checked at different concentrations of the heme analog. The results, as shown in Fig. 6d, exhibit decrease in peroxidase activity in the presence of TPPS_4_.

**Table 2:** Table generated by redox-sensitive cysteine prediction server (https://biocomputer.bio.cuhk.edu.hk/RSCP) for cysteines in DC25 sequence: The first column represents the position of cysteines, the second column represents the flanking sequences, the third column depicts the predictions and the fourth column represents the decision values.

Cysteine position	Flanking sequence	Prediction	Decision value
21	SRAYEVYFFECNKYVRVYYTP	Yes	0.80
64	GTAFAEPGIDCSFDTEASEAY	Yes	0.78
81	SEAYVFSGSQCAYIDYAPGTT	Yes	0.72
218	KVRPILDGWPCLRDILPTXXX	No	0.68

## Discussion

Legume seeds are well known for huge storage of proteins in their cotyledons. In addition to providing nutrition to the developing embryo during seed germination, many of them play important role in different metabolic processes. The water-soluble 2S albumins are among the most abundant seed proteins, some of which are rich in sulphur-containing amino acids. However, DC25 and other homologous proteins^28^ have no disulphide bonds. It is believed that seed enzymes may be active early during the germination process to hydrolyze carbohydrates^29^, to act as superoxidase dismutase for relieving the germinating seeds from oxidative stress^30,31^ and as oxalate oxidase to convert dioxygen and oxalate into hydrogen peroxide and carbon dioxide^31^. In the context of discovering and characterizing other functionally relevant seed proteins, screening of *Dolichos* seed proteome was undertaken. The analysis resulted in identification of DC25, a 2S albumin homologue abundant in legumes.

DC25 was characterized to be a monomeric thermostable protein. The presence of short loops and compact packing within its fold may have been responsible for its thermostability, as in the case of many thermophilic proteins^32^. It was known that albumins persist long during seed germination indicating their possible functional roles. The β-propeller domain of DC25 has pseudo 4-fold symmetry along the central channel. It has four blades, similar to other β-propeller proteins from plant origins. However, the number of blades are highly variable in other hemopexins^33^. Each propeller blade has strongly twisted four antiparallel β-strands in a W-like fashion. The individual β-strand differs from others in the length. It is evident that the innermost β-strands are more regular than the outer ones. The N and C termini are very close through the non-polar interactions. Due to high-resolution electron density map and protein fingerprinting, entire amino acid sequence of DC25 could be resolved. The inner side of the central channel is lined by hydrophilic residues, whereas the outer side is lined by hydrophobic residues.

The crystal structure of DC25 has three monomers in an asymmetric unit unlike other 2S albumins; CP4 has two monomers^23^, LS24 has spermine-bound/unbound dimers^24^ and CAL has a homodimer^25^. DC25, being homologous to CP4, LS24 and CAL, shows significant conformational differences in the loops connecting the outermost β-strands between the blades. They also differ on surface charge distribution which influences the quaternary structure as well as the specificity of ligand binding and function.

The presence of conserved waters among homologous proteins has some structural and functional roles. In continuation of this, conserved water molecules were determined between the blades of hemopexins from seeds. It seems they act as structural glue to tether one blade to the next by maintaining the overall tertiary structure as they function between the Ω loop and the rest of the class A β-lactamase protein structure^34^. During comparison of channels of DC25, CP4, LS24 and CAL, it was found that DC25 consists of only water molecules unlike ions. Basically, the presence of water in the channel involved in proton transfer through the formation of a protonated protein-bound water clustering as found in cytochrome C responsible for its peroxidase activity^35,36^.

Being a hemopexin, DC25 binding to a heme analog with physiological affinity was not surprising. The model of DC25 with TPPS_4_ depicts the hydrogen bonding and hydrophobic interactions, where the ligand sits comfortably in the binding pocket lined with mainly negatively charged residues. It has been reported that the loops at the narrow end of the channel are closely arranged as they would be engaged in ligand or substrate binding and catalysis^33^. The hemopexin-like domain containing proteins are present from prokaryotes to complex organisms such as plants and mammals. They perform different enzymatic activities as transferases^37^, hydrolases^38–40^, lyases^41^, isomerases^42^, and oxidoreductases^27^ in the cell. One oxidoreductase having propeller domain from *Methylophilus* is reported but there is no such protein known in plants. For the first time, we report DC25 being a plant origin oxidoreductase having peroxidase activity.

The effect of TPPS_4_ binding leading to loss in peroxidase activity suggested overlap of the ligand binding site and the active site. Combining the ligand docking results and alkylation of cysteine with iodoacetamide, Cys21 being the reactive site residue for the peroxidase activity are consistent with this observation. The single cysteine for peroxidase activity is a characteristic for 1-cysteine Peroxiredoxins (Prxs) which have conserved motif (T[P/S][V/I]]C[T/S]TE). However, this conserved motif does not exist in DC25. Hence, the DC25 may represents new mode that differs from the so-called 1-Cys Prx. Its cysteine may ionize at physiological pH that generates the thiolate group which is further oxidized by hydrogen peroxide and produces sulfenic acid (R-SOH) which is later reduced by unknown proton donor which may be reduced-glutathione, increased during seed germination to regenerate the functional protein.

In conclusion, the high resolution structure of DC25 has provided detailed structure, role of water molecules in structural architecture. The protein adopts hemopexin fold with heme binding potential as evident from its binding to a heme analog TTPS_4_. DC25 exhibits peroxidase activity which is interfered by TPPS_4_ binding. The protein may be involved in relieving the oxidative stress during germination through the active role of a surface cysteine. Indeed, the protein plays an important role in overcoming oxidative stress during seed germination.

## Materials and Methods

### Fractionation of seed proteome

*Dolichos* seeds were procured from the National Seeds Corporation, Indian Agricultural Research Institute (IARI), New Delhi. The seeds weighing 125 grams were ground and defatted that was used for protein extraction in 50 mM Tris-HCl at pH 7.5 containing 150 mM NaCl and protease-inhibitor cocktail (Sigma, P-9599) by stirring for 4 h at 4 °C. The resultant crude extract was centrifuged at 10,000 rpm for 60 min at 4 °C. The proteins were partially purified using ammonium sulfate fractionation in a range of concentrations 20–95% (w/v). The protein pellets were redissolved in 50 mM Tris-HCl buffer at pH 7.5 and the ammonium sulfate fractions were analyzed on 15% SDS-PAGE.

### N-terminal sequencing

The dominant protein bands from ammonium sulfate fractions were transferred on a polyvinylidenedifluoride (PVDF) membrane using 10 mM CAPS buffer (pH 11.0). The protein bands from PVDF were used for N-terminal sequencing by the Edman degradation method on a PPSQ-33A protein sequencer (Shimadzu Biotech). The obtained N-terminal sequences were analyzed using the BLAST algorithm^43^ in order to identify the homologous proteins.

### Protein fingerprinting and database searches

Proteins which could not be succeeded through N-terminal sequencing that were analyzed through mass spectroscopy using in-gel and in-solution digestions. The samples for protein fingerprinting were prepared using in-gel trypsin digestion method^44^ and were spotted with α-cyano-4-hydroxycinnamic acid (CHCA) matrix on matrix-assisted laser desorption/ionization (MALDI) target plate (AB SCIEX). The spots were left for drying at room temperature. Later, the laser was used to ionize the sample.

The protocol by sigma-aldrich was followed for Gluc-C in-solution digestion. The digested product was reconstituted, mixed with CHCA matrix and spotted similarly as done in case of in-gel trypsin digestion. Mass spectral analysis of the trypsin-digested and Gluc-C peptides was done using MALDI TOF-TOF 5800 instrument (AB SCIEX). The MS/MS data were processed in ProteinPilot Software 4.0 using Paragon Method where NCBInr database was used and Detected Protein Threshold was kept at 0.47.

### Protein purification

The 95% ammonium sulfate fraction was dialyzed against 50 mM Tris-HCl pH 7.5 at 4 °C. Later, the fraction was centrifuged, and the resulting supernatant was run through a weak anion exchange MonoP column (GE Healthcare), which was pre-equilibrated with 50 mM Tris-HCl pH 7.5. DC25 was eluted using a gradient of 0 to 300 mM NaCl in 50 mM Tris-HCl, pH 7.5 for 50 min. The eluted samples were evaluated on SDS-PAGE and pooled according to the purity of DC25. The pooled sample was concentrated using ultra centrifugal filters (Amicon, 10 kDa, Millipore).

### Protein characterization by mass spectrometry

Intact mass of the purified protein was determined by matrix-assisted laser desorption/ionization time-of-flight mass spectrometry (MALDI-TOF MS) with the help of an AB SCIEX TOF/TOF 5800 system. The DC25 protein in 50 mM Tris-HCl, pH 7.5 containing 60 mM NaCl buffer was mixed with sinapinic acid. The supernatant was spotted onto the MALDI target plate (AB SCIEX) and the spots were allowed to dry at ambient temperature. Later, the laser intensity of 6000 was used for analysis.

### Native gel electrophoresis (Native-PAGE)

The samples consisting of native and 30-second heat treated DC25 were run on 10% native PAGE in non-denaturing and non-reducing conditions. The running buffer was prepared by adding 0.25 M glycine and 25 mM Tris-HCl, pH 8.3. The same buffer containing 10% glycerol and 0.1% bromophenol blue was used with protein in 1:1 (v/v) to prepare samples. The gel was run at 10 mA at 4 °C.

### Thermal shift assay

The purified DC25 was diluted in 10 mM Tris-HCl, pH 8.0 using 5000X SYPRO Orange dye (Invitrogen) to give a final concentration of 10 μM protein and 10X dye in each well of PCR plate (Applied Biosystems) in a total volume of 50 μL. Hampton Stock Options pH Buffer kit (pH range of 2.2–11.0) and NaCl were added to give 50 mM buffer and 1 M, 500 mM and 60 mM NaCl in each well. The PCR plate was sealed with optically clear adhesive and centrifuged. Thermal scanning (25 °C to 95 °C) was done at an increment of 1 °C with 2 min hold using Applied Biosystems (StepOnePlus) real-time PCR.

### Crystallization, data collection and processing

The 11 mg/mL concentration of purified DC25 has been used for crystallization trials using crystal-screening kits at 4 °C with the help of an automated liquid-handling system (Mosquito, TTP labtech). The initial hit of crystallization was optimized using various precipitants, concentration, pH and temperature. The obtained single crystal was frozen along with 30% glycerol as a cryoprotectant in liquid nitrogen. Data were collected using a wavelength of 0.88560 Å with oscillation range 0.25° on the BM14 beamline at the European Synchrotron Radiation Facility (ESRF) in Grenoble, France. The program HKL2000 suite^22^ was used to process and scale the data.

### Structure determination and refinement

The mass spectrometry generated internal peptides of DC25 showed homology with hemopexin-fold protein CP4 (PDB ID: 3OYO) from cowpea through BLAST algorithm^43^. Therefore, it had been used for molecular replacement as the starting model in PHASER-MR (Simple interface) of PHENIX^45^. Further, the model was built using COOT^46^ and initial refinements were done with phenix.refine but later, restrained refinements were done using anisotropic temperature factors and local non-crystallographic symmetry (NCS) restraints in REFMAC5^47^. The developed electron density and a consensus sequence generated through multiple sequence alignment were used during model building. The analysis of DC25 structure was done using PyMOL^26^. The Polder Maps in PHENIX^45^ was used to calculate the polder omit map using reflection file and the pdb of DC25 where residues having ambiguities were omitted and solvent molecules were excluded.

### Binding affinity by surface plasmon resonance

The binding analysis of DC25 with TPPS_4_ (Alfa Aesar) was performed using a Biacore T200 system (GE) for which CM5 (carboxymethylated)-certified grade sensor chip was used to immobilize DC25 using an equal mixture of N-ethyl-N-(dimethylaminopropyl) carbodiimide and N-hydroxysuccinimide in 10 mM sodium acetate buffer at pH 4.5. The immobilization of DC25 was done at approximately 7636 RU using the protein at a concentration of 20 µM. Later, the non-reacted activated sites were blocked with 1 M ethanolamine. The reference surface was treated similarly, except that no protein was passed over it. The 1X PBS buffer was used as a binding buffer at 25 °C. The association phase was performed using various concentrations of TPPS_4_ (between 0.1 µM to 12.5 µM) in the binding buffer by allowing to bind with the immobilized DC25 at a flow rate of 30 µL/min. The dissociation phase was performed using binding buffer and regeneration was conducted using 50 mM NaOH. Biacore T200 evaluation software was used to determine equilibrium dissociation constant (K_D_).

### Ligand docking

The DC25 crystal structure was prepared for modeling through the Protein Preparation Wizard. The ligand TTPS_4_ was taken from Pub Chem and processed through LigPrep. The grid was prepared through Receptor Grid Generation where site was defined by specifying residues (Asn5, Asp63, Asp119 and Asp172). The ligand was docked through the Ligand Docking but the result was not satisfactory so the output of this docking was used for the standard induced fit docking which is performed by keeping the receptor as rigid and allowing ligand to freely change its conformation. The induced fit docking workflow of SchrÖdinger accomplishes this utilizing Glide and Prime programs which accounts for the conformational changes of the ligand and receptor, respectively. The DC25-TPPS_4_ complex having the most favorable binding energy was chosen for further analysis.

### Peroxidase activity

The reaction mixture is a total volume of 50 μL containing DC25 (10 μM) as a test protein prepared in 50 mM Tris-HCl, pH 8.0 containing 60 mM NaCl or lysozyme (10 μM) as a negative control prepared in the same buffer, or horseradish peroxidase (0.1 µM) as a positive control prepared in 0.1 M potassium phosphate buffer, pH 6, o-phenylenediaminedihydrochloride (OPD) at 0.5 mg/mL prepared in 0.1 M citrate phosphate buffer, pH 5 containing hydrogen peroxide (H_2_O_2_) at 0.015% were incubated for 5, 10, 15, and 30 mins at room temperature. The enzyme reaction was stopped using 25 μL of 1 N sulphuric acid and subsequently, absorbance was monitored at 490 nm on a Spectra Max i3X plate reader (Molecular Devices).

### Inhibition assays of peroxidase activity

The inhibition assays were carried out in two different ways. In the first way, DC25 (0.4 mg/mL) was treated with alkylating buffer (50 mM Tris-HCl, pH 8.0 containing 60 mM NaCl and 5 mM or 10 mM iodoacetamide) and the samples were incubated for 1 hr. The excess iodoacetamide was removed through buffer exchange with 50 mM Tris-HCl, pH 8.0 containing 60 mM NaCl at 4 °C. The protein sample was concentrated up to 0.6 mg/mL. An aliquot (50 µL) of the sample was added in the wells of a 96-well microtiter plate and mixed with 50 µL of the 3,3′,5,5′-tetramethylbenzidine (TMB) solution. After two minutes, the reaction was stopped with 50 µL of 1 N sulphuric acid. Subsequently, reading was recorded at 450 nm on the Spectra Max i3X plate reader (Molecular Devices).

In the second way, DC25 (100 μM) prepared in 1X PBS buffer was treated with various concentrations of TPPS_4_, prepared in the same buffer, with protein in ratios of 0:1, 1:1 and 2:1 for 60 min at room temperature. An aliquot (100 µL) of each sample was added to the wells of a 96-well microtiter plate and mixed with 100 µL of the TMB solution. The absorbance was monitored at 620 nm on the Spectra Max i3X plate reader (Molecular Devices) after 40 mins without stopping the reaction.

### Mass spectroscopy analysis of oxidized cysteine residues

The DC25 at a concentration of 0.4 mg/mL in 50 mM Tris-HCl, pH 8.0 containing 60 mM NaCl was incubated with or without 10 mM iodoacetamide at room temperature for 1 hr. After incubation, buffer exchange of the sample was done to remove excess iodoacetamide. The samples were analyzed using TOF MS method in Triple TOF 6600 system to know the number of oxidized cysteine residues after analyzing the difference between the molecular weight of alkylated and not alkylated DC25.

## Electronic supplementary material

Electronic Supplementary Material
