# Publisher Correction: High resolution structural and functional analysis of a hemopexin motif protein from *Dolichos*

**DOI:** 10.1038/s41598-020-59866-8

**Published:** 2020-02-25

**Authors:** Sarita Chandan Sharma, Ashish Kumar, Sharad Vashisht, Dinakar M. Salunke

**Affiliations:** 1 Regional Centre for Biotechnology, NCR Biotech Science Cluster, Faridabad, 121001 India; 2 Manipal Academy of Higher Education, Madhav Nagar, Manipal, Karnataka 576104 India; 3 International Centre for Genetic Engineering and Biotechnology, New Delhi, 110067 India

Correction to: *Scientific Reports* 10.1038/s41598-019-56257-6, published online 27 December 2019

A supplementary file containing Figures S1, S2 and S3 and Table S1 was omitted from the original version of this Article. This has been corrected in the HTML and PDF versions of the Article.

