# Supplementary material for: High resolution structural and functional analysis of a hemopexin motif protein from Dolichos
Source: Sci Rep. 2019 Dec 27;9:19828. doi: 10.1038/s41598-019-56257-6 (PMC6934871; doi:10.1038/s41598-019-56257-6)
Supplement: Supplementary file 1 — Electronic Supplementary Material [file 41598_2019_56257_MOESM1_ESM.pdf]

High resolution structural and functional analysis of a hemopexin motif protein from *Dolichos*

Sarita Chandan Sharma[1, 2], Ashish Kumar[1], Sharad Vashisht[1] and Dinakar M. Salunke[3,*]

[1]Regional Centre for Biotechnology, NCR Biotech Science Cluster, Faridabad-121001, India;

[2]Manipal Academy of Higher Education, Madhav Nagar, Manipal, Karnataka-576104, India;

[3]International Centre for Genetic Engineering and Biotechnology, New Delhi-110067, India.

[*]Corresponding author: Dinakar M Salunke (dinakar.salunke55@gmail.com)

Supplementary Figure Legends

Supplementary Figure S1. Biophysical characterization of DC25. Thermal shift assay to test the stability of DC25 in different concentrations of NaCl at different pH.

Supplementary Figure S2. Packing diagram of the three molecules of DC25. The supercell has been displayed in stereo with two unit cells arranged on top of each other where molecule A (green), molecule B (red) and molecule C (blue) were shown in ribbon view. The figure was prepared using PyMOL[26] (The PyMOL Molecular Graphics System; http://www. pymol. Org).

Supplementary Figure S3. The presence of monomers in one asymmetric unit and presence of conserved waters in hemopexin plant proteins. (a) Stereo view of the presence of three monomers in one asymmetric unit. (b) The presence of conserved water molecules among three monomers in asymmetric unit has been shown. (c) The conserved water molecules in all four structures (DC25, CP4, LS24 and CAL) are shown in different colors. The protein model shown here belongs to PDB ID: 6IX1. The figure was prepared using PyMOL[26](The PyMOL Molecular Graphics System; http://www. pymol. Org).

Supplementary Figure S1

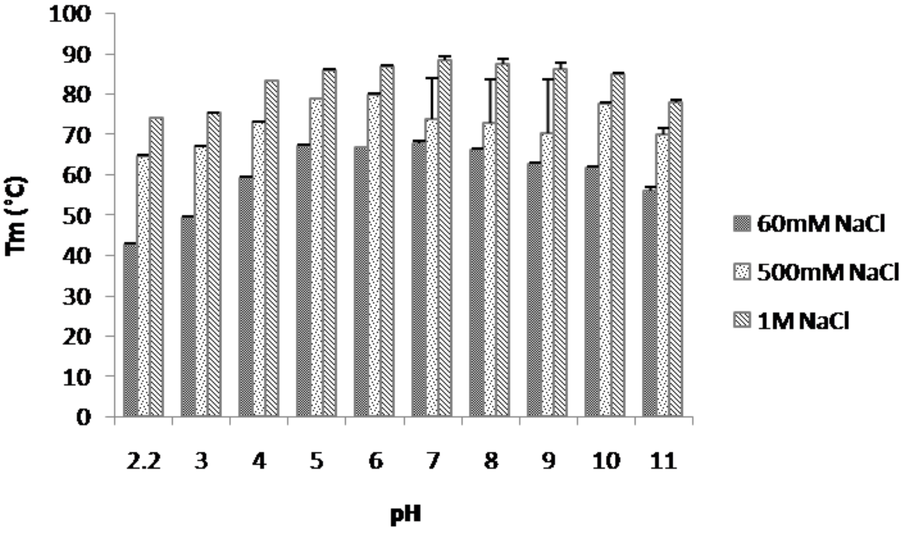

Supplementary Figure S2

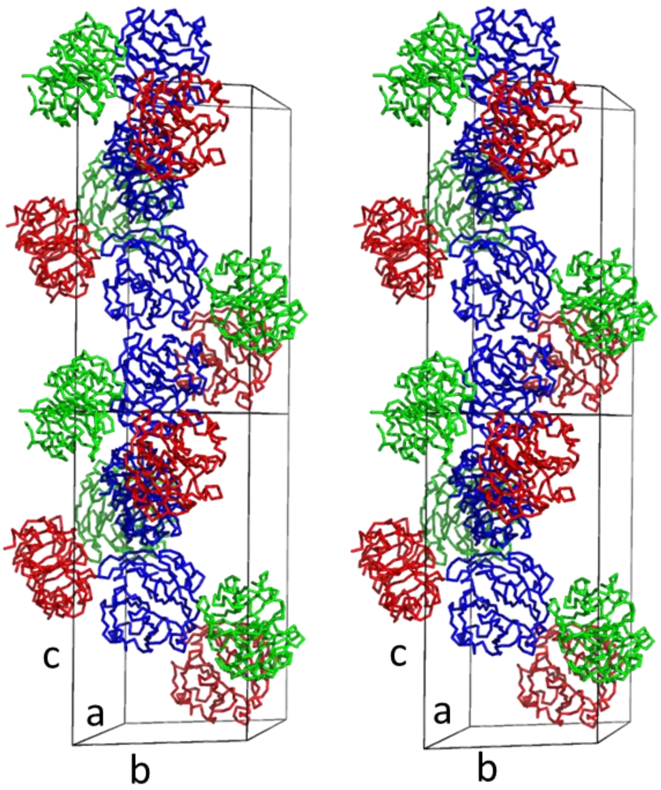

Supplementary Figure S3

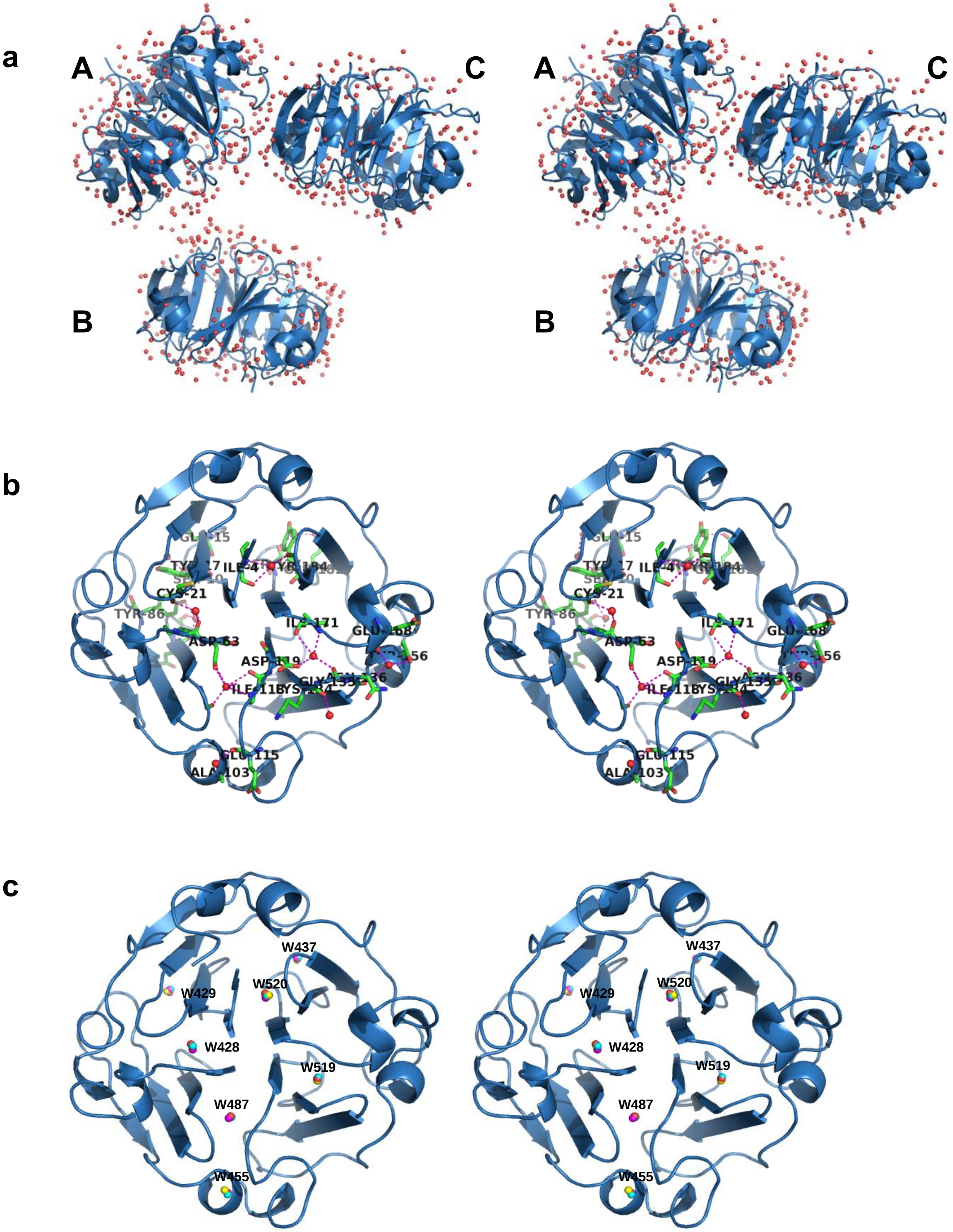

Supplementary Table S1. Identification of abundantly present proteins in *Dolichos* seeds.

**Segregated protein**	**Method of identification**	**N-terminal/Internal sequences**	**Homologous proteins**
Band 1	N-terminal sequencing	IVHRGHQESDEDSDXGGQNN	Vicilin [*Vigna unguiculata*]
Band 2 (DC25)	Mass spectrometry	SNLPYINAAFR, LQYTPGKTEDKILTNLR, ILAGPTTIAEMFPVLRNTVFADSIDSAFR, GKEVYLFK, EPEAYLFKGDKYVR （determined through trypsin digestion） SAFRSTKGKE, ESGIDASFASHKEPEA (determined through Gluc-C digestion)	Mung bean seed albumin
Band 3	Mass spectrometry	QHGTTGGFTGDTGRQHGTTGGFTGDTGR, QHGTTGGFTGDTGR, RQHGTIGDTGR, QYGTTGGFTGDTGR (determined through trypsin digestion)	Dehydrin [*Vigna unguiculata*]
Band 4	N-terminal sequencing	KAASSNVVVVEFDTYYN	Mannose lectin [*Lablab purpureus*]
Band 5	N-terminal sequencing	AQSLSFSFTKFDPNQEDLIF	A legume lectin that delays hematopoietic progenitor maturation
